# Disappearance of GFP-Positive Hepatocytes Transplanted into the Liver of Syngeneic Wild-Type Rats Pretreated with Retrorsine

**DOI:** 10.1371/journal.pone.0095880

**Published:** 2014-05-05

**Authors:** Hiromichi Maeda, Masatoshi Shigoka, Yongchun Wang, Yingxin Fu, Russell N. Wesson, Qing Lin, Robert A. Montgomery, Hideaki Enzan, Zhaoli Sun

**Affiliations:** 1 Department of Surgery, Johns Hopkins University School of Medicine, Baltimore, Maryland, United States of America; 2 Department of Surgery, Kochi Medical School, Nankoku, Kochi, Japan; 3 Cancer Treatment Center, Kochi Medical School, Nankoku, Kochi, Japan; 4 Diagnostic Pathology, Chikamori Hospital, Kochi, Kochi, Japan; National University of Singapore, Singapore

## Abstract

**Background and Aim:**

Green fluorescent protein (GFP) is a widely used molecular tag to trace transplanted cells in rodent liver injury models. The differing results from various previously reported studies using GFP could be attributed to the immunogenicity of GFP.

**Methods:**

Hepatocytes were obtained from GFP-expressing transgenic (Tg) Lewis rats and were transplanted into the livers of wild-type Lewis rats after they had undergone a partial hepatectomy. The proliferation of endogenous hepatocytes in recipient rats was inhibited by pretreatment with retrorsine to enhance the proliferation of the transplanted hepatocytes. Transplantation of wild-type hepatocytes into GFP-Tg rat liver was also performed for comparison.

**Results:**

All biopsy specimens taken seven days after transplantation showed engraftment of transplanted hepatocytes, with the numbers of transplanted hepatocytes increasing until day 14. GFP-positive hepatocytes in wild-type rat livers were decreased by day 28 and could not be detected on day 42, whereas the number of wild-type hepatocytes steadily increased in GFP-Tg rat liver. Histological examination showed degenerative change of GFP-positive hepatocytes and the accumulation of infiltrating cells on day 28. PCR analysis for the GFP transgene suggested that transplanted hepatocytes were eliminated rather than being retained along with the loss of GFP expression. Both modification of the immunological response using tacrolimus and bone marrow transplantation prolonged the survival of GFP-positive hepatocytes. In contrast, host immunization with GFP-positive hepatocytes led to complete loss of GFP-positive hepatocytes by day 14.

**Conclusion:**

GFP-positive hepatocytes isolated from GFP-Tg Lewis rats did not survive long term in the livers of retrorsine-pretreated wild-type Lewis rats. The mechanism underlying this phenomenon most likely involves an immunological reaction against GFP. The influence of GFP immunogenicity on cell transplantation models should be considered in planning in vivo experiments using GFP and in interpreting their results.

## Introduction

The accumulation of green fluorescent protein (GFP) in cells is widely used as a molecular tag that can be readily visualized under ultraviolet light illumination. Many different GFP-transgenic (Tg) animals have been generated and utilized for tracking cells in organ and cell transplantation studies. GFP can show weak immunogenicity [1], [2] and/or cell toxicity [3], [4] that can potentially alter experimental results. Gambotto et al. [1] showed that GFP could generate an antigenic epitope that binds to H2-K^d^ molecules in BALB/c mice, while Inoue and colleagues [2] generated the GFP-Tg Lewis rat (Major Histocompatibility complex haplotype; RT1^l^) and reported that transplanted skin grafts from these rats to wild-type Lewis rats lost viability after about a week, suggesting immunological rejection. Nevertheless, isolated cells from GFP-Tg rats were observed long after cell transplantation into immune-privileged sites such as the central nervous system and joints [2], [5]. In addition, liver harvested from a GFP-Tg Lewis rat survived long term in a wild-type Lewis rat without the use of an immunosuppressant (our unpublished data). These experimental findings imply that GFP is weakly immunogenic, but that organs or cells expressing GFP can survive at sites where there is a weaker immunological reaction.

In general, transplanted allogeneic hepatocytes are eliminated within a few days without the use of an immunosuppressant [6]. Nevertheless, studies with rat models suggest that GFP is minimally immunogenic when GFP-positive hepatocytes or stem/progenitor cells are transplanted into syngeneic liver. Oertel and colleagues [7] transplanted hepatocytes transfected with the GFP gene into retrorsine-pretreated wild-type syngeneic rat liver. They demonstrated continuous GFP expression, driven by the liver-specific albumin enhancer/promoter, in transplanted hepatocytes up to four months after transplantation. Other studies showed repopulation of injured liver tissue by transplanted syngeneic stem/progenitor cells expressing GFP [8], [9]. Therefore, we expected to see long-term survival of GFP-positive hepatocytes after transplantation into a wild-type Lewis rat liver.

In a pilot study, we did not observe proliferation of GFP-positive hepatocytes at six weeks after transplantation of a syngeneic liver specimen. This observation was considered to be important not only for the interpretation of previous data, but also in planning of future experiments using the rat model containing GFP-positive hepatocytes. Therefore, further studies were performed to answer three questions. 1) Did a technical error occur that prevented proliferation of GFP-positive hepatocytes? 2) Was there a loss of GFP-positive hepatocytes or a loss of GFP expression? 3) Was this phenomenon caused by a host immunological response or by GFP toxicity?

## Methods

### Animals

GFP-Tg Lewis rats, originally generated by Eiji Kobayashi [2], were obtained from the National Institutes of Health (NIH)-funded Rat Resource and Research Center, University of Missouri, Columbia, MO, USA. Male wild-type Lewis rats were purchased from Harlan Sprague-Dawley (Indianapolis, IN, USA) or from Charles-River Laboratories (Wilmington, MA, USA). Animals were maintained in the specific pathogen-free facility of the Johns Hopkins Medical Institutions and were cared for according to NIH guidelines and under a protocol approved by the Johns Hopkins University Animal Care Committee. Rats weighing 150–200 g were used as hepatocyte donors. Recipient rats weighting 180–200 g received an initial treatment of retrorsine as described below. General anesthesia during animal procedures was provided using isoflurane supplied by an isoflurane vaporizer at a concentration of 4% for induction and approximately 2% for maintenance. The muscle tone and respiratory rate of rats were closely monitored to maintain the appropriate depth of anesthesia.

### Hepatocyte isolation

**Figure 1:**
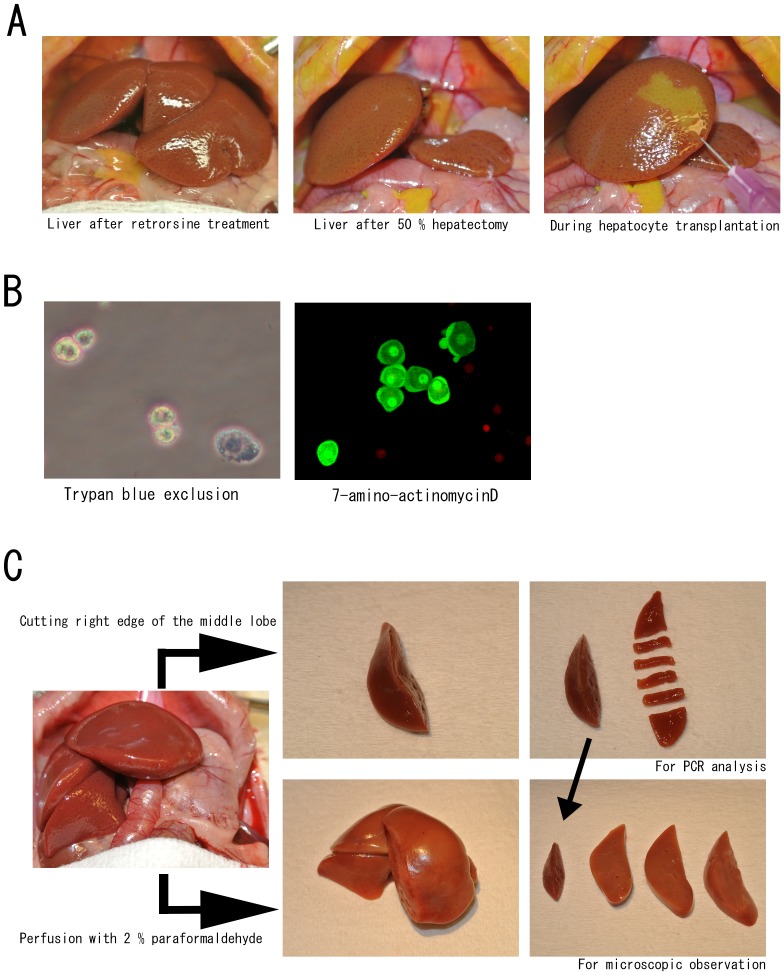
Hepatocyte transplantation and sample preparation. (A) Hepatocyte transplantation. After retrorsine treatment, the parenchyma of the liver became slightly hard and the surface was irregular. After resection of the left lobe and the left side of the middle lobe, hepatocytes were transplanted directly into the liver. The suspended cells spread rapidly according to the blood flow and produced a demarcation line, which disappeared immediately after injection. (B) Isolated hepatocytes. Trypan blue staining identified the viable hepatocytes, which were negative for 7-AAD (red stain) and expressed GFP. GFP-positive hepatocytes lose GFP expression immediately after cell death. The isolated hepatocytes cells were not completely separated. (C) Sample collection. Two weeks after cell transplantation, the remnant liver grew enlarged and swollen. (Upper arm) A small piece of the liver was obtained from the right edge of the middle lobe before perfusion from the portal vein, and then cut into smaller pieces for PCR analysis. The larger part of the liver was fixed with 2% paraformaldehyde for microscopic observation. (Lower arm) The liver was perfused with 2% paraformaldehyde and removed. The right part of the middle lobe was cut into three pieces and further processed for microscopic observation.

Hepatocytes were isolated using a two-step collagenase perfusion procedure [10] with minor modifications. Rats were sacrificed under general anesthesia by exsanguination from the inferior vena cava. The liver was immediately perfused for 10 min with pre-warmed (37°C) first solution, containing 150 mL of HBSS without calcium, 75 mg of EDTA, and 1 mL of 1 M HEPES. At the same time, the portal vein to the left lobe and the left side of the middle lobe of the liver was ligated to reduce the perfusion area. The liver was then perfused for 10 min with pre-warmed (37°C) second solution, containing 90 mL of HBSS with calcium, 10 mL of fetal bovine serum (FBS), 1 mL of 1 M HEPES, and 80 mg of collagenase type IV. The liver was transferred to a dish containing 50 mL of cold HBSS containing 10% FBS. The serous membrane was gently torn with forceps and cells that flowed out were collected. The cells were then transferred to a 50 mL tube after passing through a 70-µm mesh filter without mechanical pressure, and the collected cells were centrifuged at 50×*g* for 1 min. The supernatant containing non-parenchymal cells was discarded and the pellet was washed by resuspension in 50 mL of HBSS containing 10% FBS, and then centrifuged. This washing step was repeated. The cell number and viability were calculated and the cell density was adjusted to 1.5×10^7^ cells/mL for cell transplantation and 1×10^8^ cells/mL for immunization (Fig. 1). The viability of hepatocytes was maintained at more than 80% throughout the experiments. One donor provided hepatocytes for between four and six recipient rats.

### Hepatocyte transplantation

The livers of rats were accessed by laparotomy under general anesthesia. The left lobe and right side of the middle lobe of livers were removed to achieve approximately 40–50% hepatectomy (50% hepatectomy) [11]. A 28 G needle was inserted deep into the right side of the middle lobe and the hepatocytes were injected with the needle slowly being pulled back. This procedure was repeated three times using different puncture sites, allowing the transplanted hepatocytes to distribute throughout the whole right side of the middle lobe. This method prevented embolic injury to the other hepatic lobes and enabled us to safely perform a core needle biopsy one week after cell transplantation. After 1×10^7^ cells in total were transplanted, the hemostasis was checked and the wound was closed.

### Preparation and administration of retrorsine

Retrorsine treatment was used to inhibit the mitosis of mature hepatocytes and to facilitate the proliferation of transplanted hepatocytes. A 30-mg/kg of retrorsine was administered into the peritoneal cavity of recipient rats twice, two weeks apart, according to the original method [12]. The retrorsine dose was prepared by adding 100 mg of retrorsine into 10 mL of normal saline, whereupon 1 M HCl was added until the solution reached pH 2. Once the retrorsine was completely dissolved, the solution was neutralized with 1 M NaOH. The retrorsine dose was freshly prepared and used immediately for each treatment.

### Sample preparation

After laparotomy under general anesthesia, the right edge of the middle liver lobe was cut with a surgical scalpel. Small pieces of tissue were dissected from this site for DNA analysis and the remaining small piece of tissue was fixed in 2% paraformaldehyde for microscopic observation (Fig. 1). After exsanguination, the liver was slowly perfused with 10 mL of cold PBS and then with 30 mL of 2% paraformaldehyde. After excision of the whole liver, the right upper lobe was cut into three pieces and further fixed with 2% paraformaldehyde for 30 min at room temperature in the dark. The liver samples were then immersed in a 30% sucrose solution and stored at 4°C overnight, before embedding in OCT compound (Sakura Finetek USA, Torrance, CA, USA) and storage at −80°C.

### Percentage area occupied by transplanted hepatocytes in recipient liver

Sections (6 µm thickness) were prepared from each of four liver tissue samples from each rat. Images of three random fields from each section slide were recorded, providing a total of 12 fields from each rat. The relative area of the transplanted hepatocyte area as a percentage of the total area was calculated by using Photoshop CS3 (Adobe systems, San Jose, CA, USA).

### Immunohistochemistry staining

Immunohistochemistry staining was performed to confirm the expression of GFP expression by light microscopy. The tissue sections were incubated with 1% SDS in PBS for 7 min, followed by incubation with 1% H_2_O_2_ in PBST for 60 min. The presence of endogenous biotin was blocked using an avidin-biotin blocking reagent (Avidin Blocking System; DAKO, Cambridgeshire, UK), and then sections were incubated with biotin-conjugated anti-GFP antibody (1∶200 dilution, Abcam, Cambridge, MA, USA) for 1 h. Tissue sections were then incubated with AB complex (VECTASTAIN ABC kit; Vector, Burlingame, CA, USA) for 30 min according to the manufacturer's instructions. The signal was detected with DAB (Liquid DAB+ Substrate Chromogen System; DAKO), and the tissue sections were counterstained with haematoxylin for 20 s.

### Immunofluorescent staining

The expression of albumin by the transplanted hepatocytes was detected using immunofluorescent staining. The tissue sections were pre-incubated with 1% SDS in PBS for 12 min, whereupon they were incubated with sheep anti-rat albumin primary antibody (1∶800 dilution, Bethyl laboratory Inc, Montgomery, TX, USA) for 30 min. The sections were then washed and incubated with Cy3-conjugated donkey anti-sheep IgG secondary antibody (1∶400 dilution, Jackson Immunoresearch Laboratories, West Grove, PA, USA) for 30 min.

The phenotype of infiltrating cells adjacent to GFP-positive transplanted hepatocytes was investigated by assaying the expression of CD4, CD8, and ED-2. The tissue sections were pre-incubated with 1% SDS in PBS for 1–2 min. CD4 was detected using goat anti-CD4 primary antibody (1∶100 dilution, Santa Cruz Biotechnology, Dallas, TX, USA) and Cy3-conjugated donkey anti-goat IgG antibody (1∶100 dilution, Jackson Immunoresearch Laboratories). CD8 was detected using rabbit anti-CD8 primary antibody (1∶100 dilution, Santa Cruz Biotechnology) and Cy3-conjugated donkey anti-rabbit IgG secondary antibody (1∶100 dilution, Jackson Immunoresearch Laboratories). ED-2 was detected using mouse anti-ED-2 primary antibody (1∶100 dilution, Santa Cruz Biotechnology) and Cy3-conjugated donkey anti-mouse IgG secondary antibody (1∶100 dilution, Jackson Immunoresearch Laboratories). In order to characterize GFP-positive non-parenchymal cells, rat endothelial cell antibody (RECA) was detected by using mouse anti-RECA primary antibody ((1∶100 dilution, Abcam), and Cy3-conjugated donkey anti-mouse IgG secondary antibody.

Sections were incubated with the primary antibody for 60 min and with the secondary antibody with 30 min. Nuclear staining was performed using DAPI (DAKO) staining when necessary. Serum blocking was performed with 10% donkey serum for 10 min.

### Genomic DNA extraction and PCR analysis

Tissue samples were quick-frozen in liquid nitrogen and stored at −80°C until use. Genomic DNA was extracted by first treating the tissue with proteinase K, and then isolating the DNA by phenol-chloroform extraction (Invitrogen, Carlsbad, CA, USA). The presence of the GFP gene in each sample was analyzed using PCR with 1 µg of genomic DNA for each sample, and 0.1 µg of genomic DNA for the positive and negative controls. The PCR comprised 30 cycles of amplification. The DNA content of each sample analyzed for GFP was normalized to that of the *Rattus norvegicus* Sry gene, which is the sex-determining region on Y chromosome. The Sry copy number per haploid genome is similar among all male rat cells. The presence of the Sry gene was analyzed using 0.1 µg of genomic DNA in the samples and controls with 28 amplification cycles.

### Bone marrow transplantation

Bone marrow cells were obtained from the bilateral tibia and the femoral bone of male Lewis rats weighing 150–200 g. The bone marrow from one rat provided sufficient donor cells for one recipient rat. Erythroblasts and erythrocytes were removed using red blood cell lysing buffer (Sigma Aldrich, St. Louis, MO, USA). Bone marrow cells were washed and approximately 2–3×10^8^ cells were injected via the tail vein into each recipient rat on the same day that the recipient rat received 11 Gy of whole body irradiation. The relative numbers of GFP-positive leukocytes in peripheral blood was assayed using flow cytometry one day before hepatocyte transplantation at seven weeks after bone marrow transplantation.

### Experimental protocol

#### Experiment 1 - Confirmation of GFP-Tg cell-loss phenomenon (Figs. 2 and 3)

A total of 12 wild-type Lewis rats were obtained from Harlan Sprague-Dawley and received hepatocytes from GFP-Tg Lewis rats after retrorsine treatment and 50% hepatectomy. All rats underwent core needle biopsy of the liver seven days after cell transplantation and then were sacrificed on days 14, 28, or 42. For comparison, 12 GFP-Tg Lewis rats received similarly prepared hepatocytes from wild-type Lewis rats. The same studies were performed using the Lewis rats obtained from the Charles River Laboratories and liver samples were collected on days 14 (n = 3) and 42 (n = 3).

**Figure 2:**
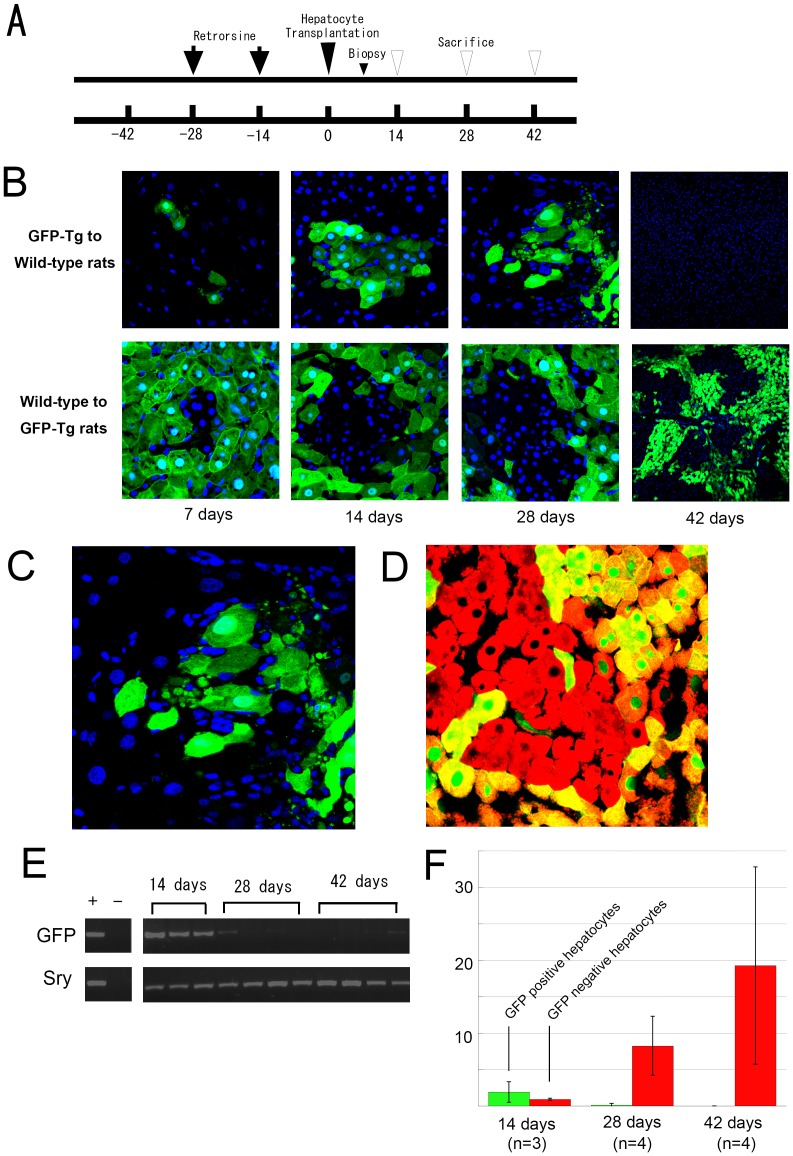
Disappearance of GFP-positive hepatocytes in wild-type syngeneic rat liver. (A) Timeline of Experiment 1. Recipient rats received retrorsine treatment twice (black arrow). One week after hepatocyte transplantation (large black triangle), all rats had a liver biopsy under general anesthesia, and then rats were sacrificed on days 14, 28, or 42 (white triangle). (B) Confocal microscopic observation of GFP fluorescence in hepatocytes after transplantation into wild-type Lewis rats. (Upper lane) The liver biopsy taken at day seven shows engraftment of transplanted hepatocytes in all cases. The nodules of GFP-positive hepatocytes after transplantation were less numerous at day 28 than at day 14. GFP-positive hepatocytes at 28 days also showed more heterogeneous GFP expression with an irregular cell shape than at day 14, indicating cytoplasmic degenerative changes. The dissociation of GFP-positive hepatocytes was apparent along with nuclear debris (small green dots). The infiltration of GFP-negative cells with a small nucleus (blue stain) was also apparent. (Lower lane) The progressive growth of GFP-negative hepatocytes in GFP-Tg Lewis rat liver resulted in the size of each cluster of transplanted hepatocytes increasing with time. (C) Higher magnification of the liver tissue at 28 days after hepatocyte transplantation from GFP-Tg rats to wild-type rats. (D) Merged image of immunofluorescent staining for albumin (red) and immunohistochemical staining for GFP (green). The transplanted hepatocytes from wild-type rats proliferate and express albumin in GFP-Tg Lewis rat liver. (E)The percentage of transplanted GFP-positive hepatocytes increased by 14 days after cell transplantation, but significantly decreased by 28 days, and no GFP-positive hepatocytes were observed at day 42. In contrast, the percentage of GFP-negative transplanted hepatocytes increased steadily. (F) PCR analysis of the GFP transgene showed a reduction at 28 and 42 days, suggesting elimination of the GFP transgene.

**Figure 3:**
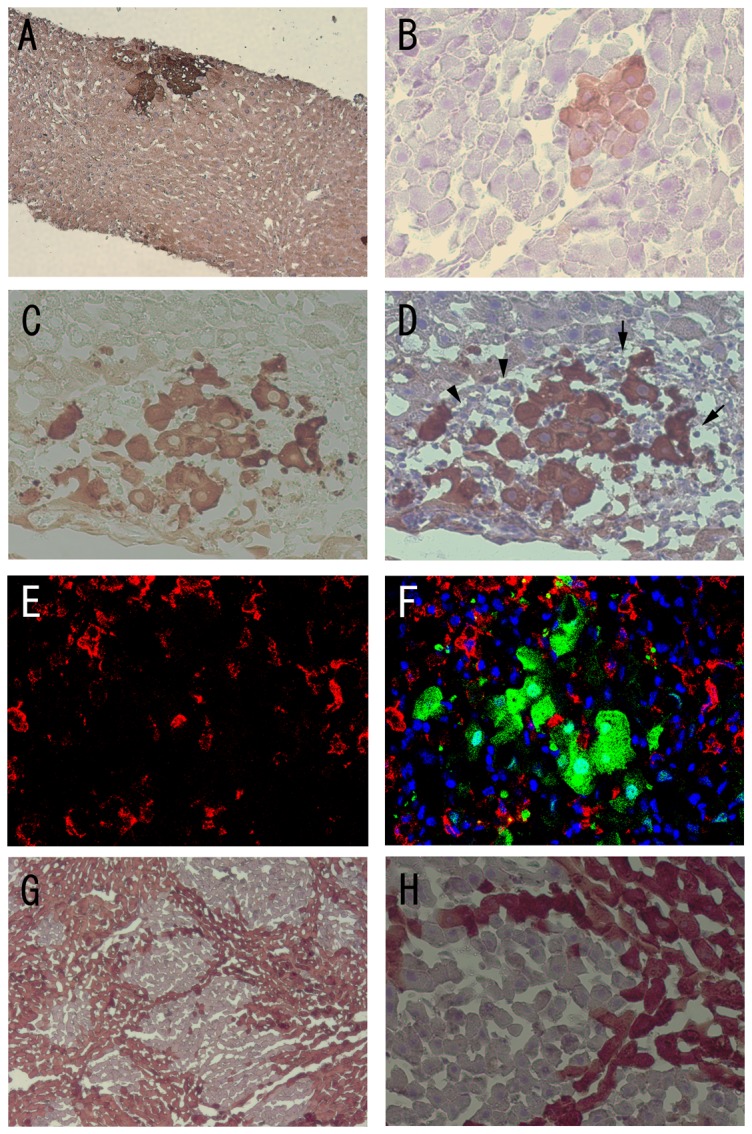
Immunohistochemical staining for GFP and immunofluorescent staining for ED-2. (A), (B) The liver specimen showed small clusters of GFP-positive hepatocytes at 7 and 14 days after hepatocyte transplantations into wild-type Lewis rat liver. (C), (D) However, there were fewer GFP-positive hepatocytes apparent at 28 days after hepatocyte transplantation, and dissociated, irregular-shape polygonal cells with debris were observed. Small cells with round nuclei (lymphocytes: arrow) were abundant around the GFP-positive hepatocytes. Cells with a triangular crescent cytoplasmic shape (arrowhead) were reminiscent of Kupffer cells. (E) Some of the infiltrating cells are positive for ED-2 (red). (F) Merged image of GFP (green), ED-2 (red), and DAPI nuclear staining (blue) suggests the accumulation of Kupffer cells adjacent to GFP-positive hepatocytes. (G), (H) The transplanted GFP-positive hepatocytes continued to proliferate at 42 days after hepatocyte transplantation from GFP-negative to GFP-Tg Lewis rats.

#### Experiment 2 - Effect of an immunosuppressant on GFP-Tg cell loss (Fig. 4)

Rats were treated with the immunosuppressive drug, tacrolimus, to investigate if loss of transplanted hepatocytes is related to immunological rejection. Recipient wild-type Lewis rats received 0.3 mg/kg of tacrolimus subcutaneously for five days a week after hepatocyte transplantation, and then were sacrificed on day 42. A control group of rats received the same quantity of normal saline for five days a week.

**Figure 4:**
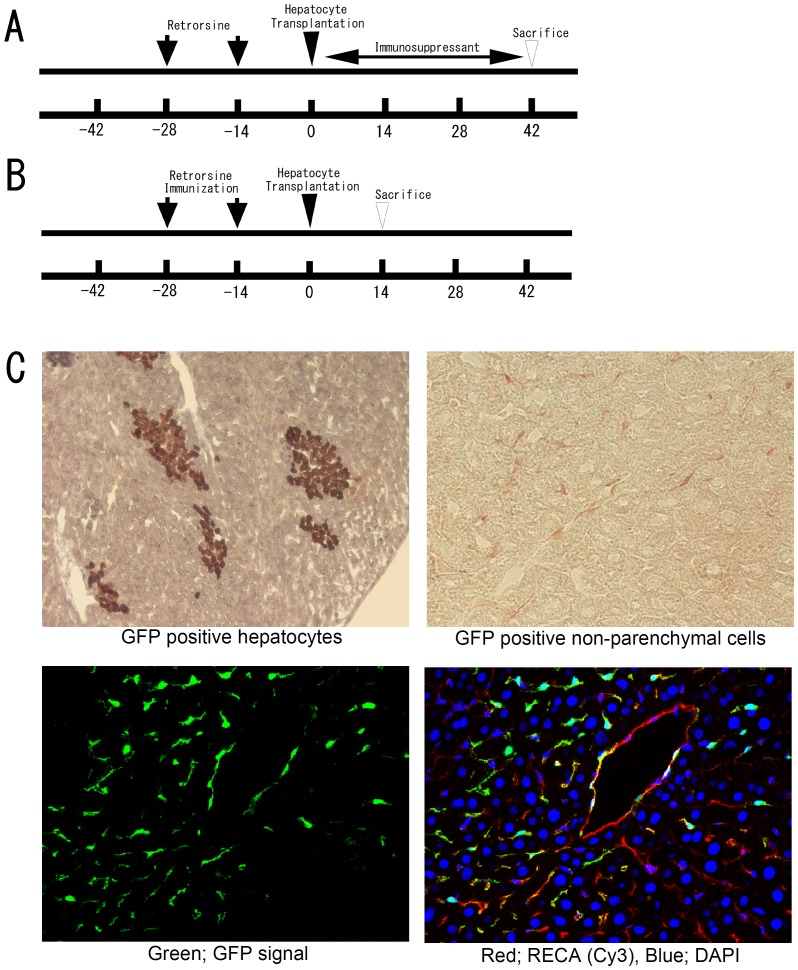
The effect of immunosuppressant or sensitization on GFP-positive hepatocyte survival after transplantation. (A) Timeline of Experiment 2. A dose of 0.3 mg/kg of tacrolimus was administered after hepatocyte transplantation for five days a week, and tissue samples were collected 42 days after transplantation. The control group received normal saline instead of tacrolimus. (B) Timeline of Experiment 3. The recipient rats received a subcutaneous injection of GFP-positive hepatocytes (1×10^8^ cells) on the same day as retrorsine treatment. Fourteen days after hepatocyte transplantation into the liver, tissue samples were collected to evaluate the survival of transplanted hepatocytes. (C) After immunosuppressant treatment, the specimen shows GFP-positive hepatocytes and abundant GFP-positive sinusoidal lining cells. These non-parenchymal cells were also positive for the RecA marker of endothelial cells.

#### Experiment 3 - Immunization with GFP-positive hepatocytes (Fig. 4)

Wild-type rats received a subcutaneous injection of 1×10^8^ isolated hepatocytes from GFP-Tg rats on the same day as retrorsine treatment. A control group of rats received hepatocytes from wild-type Lewis rats. Liver samples were collected two weeks after hepatocyte transplantation (Fig. 4).

#### Experiment 4 - Modification of the immunological reaction by bone marrow transplantation (Fig. 5)

Bone marrow transplantation was performed prior to hepatocyte transplantation to exclude any potential protective effect of tacrolimus on hepatocytes [13] and to confirm the involvement of an immunological reaction. Wild-type Lewis rats received 11 Gy of whole body irradiation and then bone marrow cells from GFP-Tg Lewis rats were transplanted, three weeks prior to retrorsine treatment. Two weeks after the second administration of retrorsine, the rats underwent partial hepatectomy and received hepatocytes from the GFP-Tg Lewis rats (Group 1). Rats in Group 2 received bone marrow transplantation from wild-type Lewis rats. Rats in Group 3 received bone marrow transplantation from GFP-Tg Lewis rats and received HBSS with FBS instead of hepatocyte transplantation. Six weeks after cell transplantation, liver samples were collected.

**Figure 5:**
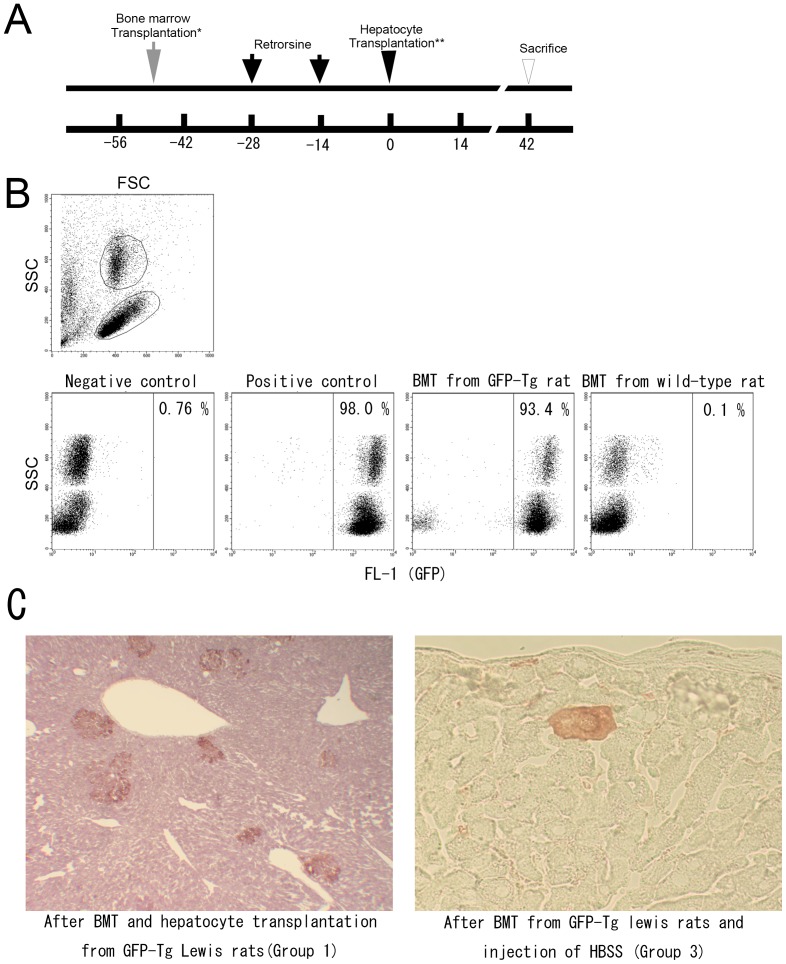
Reconstruction of immune system by bone marrow transplantation and hepatocyte transplantation. (A) Timeline of experiment 5. Wild-type recipient rats received 11Gy of whole body irradiation and bone marrow transplantation (BMT). Rats in Group 1 received bone marrow cells from GFP-Tg Lewis rats and after retrorsine treatment and hepatectomy, GFP-positive hepatocytes were transplanted and the rats were sacrificed on day 42. Rats in Group 2 received bone marrow cells from wild-type Lewis rats and GFP-positive hepatocytes (*). The rats in Group 3 received bone marrow transplantation from GFP-Tg rats and HBSS with 10% FBS instead of hepatocyte transplantation (**). (B) Flow cytometry of peripheral blood 7 weeks after bone marrow transplantation. More than 99% of leukocytes in the blood from GFP-Tg rats were GFP-positive, as compared to more than 90% of leukocytes after transplantation of GFP-positive bone marrow cells. After bone marrow transplantation from wild-type rats to wild-type rats, practically none of the leukocytes were positive for GFP. (C) Livers from Group 1 rats showed clusters of GFP-positive hepatocytes at 42 days after hepatocyte transplantation and from Group 3, a few GFP-positive large polygonal cells were observed residing within hepatic cords. These cells are similar to megalocytic GFP-positive hepatocytes [27]. Clusters of GFP-positive small hepatocyte-like progenitor cells or mature hepatocytes were not detected.

## Results

### Mortality and morbidity

One rat from each group in Experiment 1 died of injury to the inferior vena cava or injury to the small intestine during hepatectomy, respectively. No other mortality or late complications, including liver failure and infection, were observed.

### Experiment 1 - Disappearance of GFP-positive hepatocytes

All of the liver biopsies (n = 11) showed engraftment of transplanted hepatocytes from GFP-Tg rats into wild-type rats at seven days after transplantation (Fig. 2). Clusters of GFP-positive hepatocytes were larger and more frequently detected at 14 days after transplantation than at 7 days, suggesting rapid growth of the transplanted hepatocytes. At 28 days after hepatocyte transplantations, the rat livers showed very few clusters of GFP-positive hepatocytes and they exhibited cytoplasmic degenerative changes with nuclear debris (Fig. 2). These cells were surrounded by a large number of GFP-negative, small, round lymphocyte-like cells that were positive for CD4 or CD8 (results not shown). Slightly larger, ED-2-positive, triangular cells that were suggestive of Kupffer cells, were observed in the same region (Fig. 3). A small number of GFP-positive and RECA-positive non-parenchymal cells were also observed. No GFP-positive hepatocytes and only a few GFP-positive non-parenchymal cells were detected at 42 days after transplantation. In contrast, wild-type GFP-negative hepatocytes steadily increased in number after transplantation into GFP-Tg rat liver. At 42 days after transplantation, approximately 19.3% (range; 5.6%–35.2%) of the liver area was occupied by transplanted GFP-negative hepatocytes. The transplanted hepatocytes were largely evenly distributed and each liver specimen from a single rat showed similar features by direct fluorescent microscopy, which is consistent with a previous report [14].

PCR analysis for the GFP transgene in wild-type rat liver showed reduced amounts at four and six weeks after transplantation. This suggests a gradual elimination of GFP-positive cells rather than loss of GFP expression by any remaining viable transplanted cells or their progeny cells. We also examined liver specimens three months after cell transplantation from GFP-Tg Lewis rats to wild-type rats (n = 5), and observed complete loss of GFP-positive hepatocytes and non-parenchymal cells by both fluorescent microscopy and PCR analysis for the GFP transgene (data not shown). This observation was also confirmed using Lewis rats from Charles-River laboratories.

### Experiment 2 - Immunosuppressant treatment prolongs survival of transplanted GFP-Tg hepatocytes

The livers of rats treated with the immunosuppressant, tacrolimus, showed abundant GFP-positive hepatocytes and GFP-positive non-parenchymal cells at 42 days after cell transplantation. Control rats that received only saline for 5 days a week showed no GFP-positive hepatocytes at 42 days after cell transplantation (Fig. 4). Non-parenchymal cells were mainly positive for RECA by immunofluorescent staining and few inflammatory cells were found in the vicinity of transplanted hepatocytes.

### Experiment 3 - Host pre-immunization with GFP-Tg hepatocytes shortens the survival of transplanted GFP-Tg

The livers of rats treated by pre-immunization with GFP-Tg hepatocytes showed no GFP-positive hepatocytes at 14 days after cell transplantation. Cell-like structures exhibiting green fluorescence were observed in the livers, but no viable cells with GFP expression were identified, nor were GFP-positive hepatocytes detectable at the sites of subcutaneous injection of GFP-Tg hepatocytes. Control rats that were pre-immunized with wild-type hepatocytes showed clusters of GFP-positive hepatocytes at 14 days after cell transplantation.

### Experiment 4 - Modification of the immunological reaction by bone marrow transplantation

More than 90% of leukocytes in the peripheral blood of wild-type rats were positive for GFP expression seven weeks after bone narrow transplantation from GFP-Tg Lewis rats (Fig. 5). In Group 1, GFP-positive hepatocytes continued to survive in wild-type rat liver for at least 42 days after hepatocyte transplantation. In contrast, the rats in Group 2 that received bone marrow of wild-type rats and GFP-Tg hepatocyte transplantation showed no GFP-positive hepatocytes in the liver except in one rat (n = 4). Abundant GFP-positive non-parenchymal cells were observed, which suggests incomplete reconstruction of their immune system after bone marrow transplantation that resulted in a weaker immunological reaction against transplanted cells. Rats in Group 3 received bone marrow from GFP-Tg Lewis rats and an HBSS injection instead of hepatocyte transplantation. Their livers showed a small number of GFP-positive hepatocyte-like cells that maintained their morphology and were larger than surrounding normal hepatocytes (Fig. 5). Thus, these bone marrow-derived hepatocyte-like cells were distinguishable from transplanted GFP-positive hepatocytes.

## Discussion

This study demonstrated that GFP-positive hepatocytes isolated from GFP-Tg rats could engraft in wild-type host rats. Importantly, the transplanted cells did not persist for more than 42 days in a wild-type syngeneic rat liver that was pretreated with retrorsine and by partial hepatectomy. In contrast, hepatocytes transplanted from wild-type rats steadily proliferated in GFP-Tg Lewis rat liver. Immunosuppressant treatment with tacrolimus prolonged the survival of GFP-positive hepatocytes, whereas pre-immunization with GFP-Tg hepatocytes decreased the time to disappearance of transplanted hepatocytes in wild-type rats. Prolonged survival of GFP-positive hepatocytes by bone marrow transplantation eliminated the potential protective effect of tacrolimus on GFP-Tg hepatocytes [13]. These results strongly suggest that the disappearance of transplanted hepatocytes in our model was primarily due to an immunological reaction to the GFP transgene rather than to GFP toxicity.

GFP-Tg Lewis rats were originally generated using Lewis rats obtained from Charles-River Laboratories Japan (Yokohama, Japan), the rats exported from Charles-River Laboratories in the USA in 1981 (personal communication, Charles-River Laboratories, Japan). We initially noticed the disappearance of transplanted hepatocytes by using wild-type Lewis rats from Harlan Sprague-Dawley, and hypothesized that these two rats from two different colonies might express different antigens affecting the immunological reaction. In fact, the phenomenon was reproduced in wild-type Lewis rats from Charles-River Laboratories. Therefore, we consider that the cellular loss after GFP-positive hepatocyte transplantation is due to an immunological reaction against GFP.

Our findings are consistent with the results of a GFP gene transfer study into the liver of immune competent mice [15], although they contradict a transplantation study of hepatocytes expressing GFP [7]. Follenzi et al. [16] used a lentivirus vector to introduce GFP transgenes driven by the cytomegalovirus (CMV) enhancer/promotor into hepatocytes of SCID mice. These authors used fluorescence microscopy to demonstrate the continuous and stable expression of GFP; however, the number of GFP-expressing hepatocytes decreased or disappeared in immune competent mice livers by two weeks after transplantation [15]. Notably, the kinetics of GFP-positive hepatocytes depended on the strain of the mouse, with fewer GFP-positive hepatocytes in C57BL/6 mice, and their disappearance in FVB/N and BALB/c strains within two weeks. That study measured vector DNA content in the liver, and our findings suggested that GFP-positive cells were eliminated from the liver rather than viable cells losing their GFP expression. In contrast, cultured hepatocytes and hepatoblasts with the GFP transgene driven by the albumin promoter showed vigorous proliferation and continuous GFP expression after transplantation into a syngeneic rat liver that did not express dipeptidyl peptidase-4 (DPPIV) in their liver (DPPIV-negative) and had been pretreated with the inhibitor of hepatocyte proliferation, retrorsine. The liver samples from these rats clearly showed groups of GFP-positive hepatocytes which were also positive for DPPIV in DPPIV-negative syngeneic rat liver for at least four months. The reasons for these inconsistent results are not clear as yet, but it is possible that DPPIV-negative animals produce a weak immune response against GFP. DPPIV is also expressed on the surface of T cells [17], where it might play a functional role in regulating the immune system. It is also possible that GFP is minimally antigenic in F344 rats, which were often used in previous studies [7], [8], [9].

Immunological factors that could affect the outcome of hepatocyte transplantation include conventional immunosuppressants [18], depletion of immune cells [7], and reconstruction of the immune system by bone marrow transplantation. Additional important factors that can affect an immune response include the role of stem/progenitor cells as immune modulators [20], the amount of antigen, and activation of the immune system by the antigen-presenting cells [15]. Follenzi et al. [15] reported that the absence of GFP expression in antigen-presenting cells results in longer survival of GFP-expressing hepatocytes in a mice model [15]. In our model, cell-to-cell connections were roughly digested and the presence of contaminating non-parenchymal cells in the transplanted hepatocytes was highly likely. This contamination could partially explain the difference between our results and those of Oertel et al. [7]. Severe liver injuries are often utilized to enhance the engraftment and proliferation of transplanted cells. This enhancement occurs by providing both a niche for cell proliferation and a growth stimulus to transplanted cells. In fact, the engraftment of transplanted cells and repopulation of hepatocytes occurs to a lesser extent without liver injury [19]. We also speculate that severe and fulminant liver injury can result in a reduced host capacity for immune response, thereby potentially permitting the survival of transplanted hepatocytes that would be rejected under normal conditions [21].

The characteristics of transplanted cells are important in determining the outcome of their transplantation. A previous study using a retrorsine-treated rat model demonstrated that Thy1-positive hepatocytes grew rapidly in DPPIV-negative rat livers, but disappeared within two months [22]. Under the same conditions mature hepatocytes grew slowly and steadily [22]. Furthermore, mesenchymal stem cells (MSCs) isolated from different species behaved differently. In a liver regeneration model, MSCs isolated from syngeneic rats do not differentiate into hepatocytes [14] whereas transplanted human MSCs could differentiate into functioning hepatocytes in the injured livers of rats or pigs [21], [23]. This difference might be attributable to the different properties of human and rat MSCs when human MSCs are xenotransplanted and when rat MSCs undergo syngeneic transplantation [14].

Notably, the liver from GFP-Tg Lewis rats can survive in wild-type syngeneic rats (data not shown) despite vigorous rejection of transplanted hepatocytes in this situation. Typically a liver graft is rejected approximately 10 days after allogeneic transplantation when there is a severe rejection reaction. A shorter treatment with a low dose of tacrolimus resulted in the liver graft surviving in allogeneic recipients despite the MHC barrier [24]. Generally, the same amount of immunosuppressant treatment is insufficient to prevent graft loss of other organs such as heart, kidney, and skin, and the mechanism of liver graft survival after shorter and smaller doses of immunosuppressant includes the release of non-specific immune modulators from the liver graft and the functional role of non-parenchymal cells in the graft [25], [26]. In contrast, the majority of the transplanted hepatocytes are eliminated from the host liver within the first few days by a nonspecific immune response [6] that leads to foreign antigen presentation and acceleration of the immune reaction. Identification of the major mechanism determining the tolerance of liver grafts and the rejection of hepatocytes is of major importance. Modulation of the factors involved would be beneficial for the control of immune response after liver and hepatocyte transplantation, as well as other organ transplantation.

Very few large, GFP-positive polygonal cells were residing in the hepatic cords of retrorsine-pretreated livers after bone marrow transplantation but without hepatocyte transplantation, as shown in Experiment 4. Our previous study using transplantation of wild-type retrorsine-pretreated liver into GFP-Tg syngeneic rats identified a similar group of cells that represented less than 0.02% of total hepatocytes at eight weeks after liver transplantation [27]. This population of hepatocytes expressed GFP, albumin, and CYP1A2, suggesting that they were functional hepatocytes. Majorities of these abnormally large, hepatocyte-like cells resided in hepatic cords solitarily despite the expression of Ki67. We also identified a group of small cells that were capable of vigorous proliferation in the same liver samples. Therefore, it is plausible that the abnormally large GFP-positive polygonal cells are derived from the fusion of endogenous hepatocytes exposed to retrorsine and bone marrow cells, whereas the very small polygonal cells are derived by transdifferentiation of bone marrow cells. Thus, we agree with the model proposed by Masson et al. that cell fusion and cell transdifferentiation depends upon the liver environment [28]. A comprehensive study using sex-mismatched liver or bone marrow transplantation is necessary to clarify this issue.

In this experiment, we have demonstrated that hepatocytes from GFP-Tg Lewis rats are not able to survive long-term in the syngeneic wild-type Lewis rat liver. Liver is not an immune-privileged site for hepatocyte transplantation, and multiple factors determine the death or survival of transplanted hepatocytes. It is also notable that the progression of the cell loss phenomenon observed in the current study did not alter when more severe treatment such as 2/3 hepatectomy (n = 3) and 80% hepatectomy (n = 1) with retrorsine treatment was employed. This suggests that an immunological reaction against the transplanted GFP-positive hepatocytes is maintained in this strong liver regeneration model. In conclusion, this study demonstrated the need to consider the host immunological reaction in the hepatocyte transplantation model using GFP-Tg Lewis rats as donors.
